# Microstructural Evolution and Ultrafine-Grain Formation During Flow Forming of Thick-Walled Cu–Ni Alloy Tubes

**DOI:** 10.3390/ma19142968

**Published:** 2026-07-09

**Authors:** Jie Zhao, Qinxiang Xia, Gangfeng Xiao, Delin Tang, Han Sun

**Affiliations:** 1 School of Mechanical and Automotive Engineering, South China University of Technology, Guangzhou 510641, China; 202111080674@mail.scut.edu.cn (J.Z.); meqxxia@scut.edu.cn (Q.X.); sunhan@scut.edu.cn (H.S.); 2 Yantai Wanlong Vacuum Metallurgy Co., Ltd., Yantai 264000, China; tangdelin@yt-wlvm.net

**Keywords:** flow forming, thick-walled tube, Cu–Ni alloy, microstructural evolution, ultrafine-grain

## Abstract

Flow forming has emerged as an effective route for manufacturing thick-walled Cu–Ni alloy tubes, particularly for producing gradient microstructures. To clarify the evolution of microstructure during deformation and to elucidate the mechanism governing ultrafine-grain formation, flow forming experiments were conducted on BFe10-1-1 thick-walled tubes. Finite element (FE) simulations and electron backscatter diffraction (EBSD) characterization were performed in parallel. On this basis, a coupled finite element analysis–cellular automaton (FEA-CA) microstructure evolution model was established, in which the local equivalent plastic strain (PEEQ) gradient was introduced to modify the geometrically necessary dislocation (GND) distribution at grain boundaries. The results reveal that the microstructural transformation during flow forming proceeds through a continuous high-angle boundary development pathway resembling continuous dynamic recrystallization. A marked through-thickness gradient in grain size is observed. Owing to the higher accumulated strain and stronger strain-gradient effects, the outer surface layer undergoes accelerated refinement and forms a stable banded ultrafine-grained structure with an average grain size of approximately 0.39 μm. The governing mechanism of ultrafine-grain formation exhibits a distinct pass-dependent response. During Pass 1, rapid substructure establishment dominates. In Pass 2, substantial grain refinement is driven by progressive grain-boundary misorientation increase and high-angle transformation. By Pass 3, the refinement rate decreases noticeably, and the microstructure approaches a saturated and relatively stable state.

## 1. Introduction

Thick-walled tubes, generally defined as tubular components with a thickness-to-diameter ratio exceeding 0.05, serve as critical load-bearing structures in marine engineering applications [1]. Their long-term exposure to aggressive environments—characterized by seawater immersion, biofouling, and high hydrostatic pressure—imposes stringent requirements on corrosion resistance, wear resistance, and mechanical reliability. Conventional manufacturing of thick-walled tubes typically follows a combined “casting–forging–extrusion” route. Although mature, this process chain involves multiple stages, substantial energy consumption, and significant equipment investment. Moreover, hot-working operations frequently induce grain coarsening and surface cracking, both of which compromise structural integrity and service reliability [2]. Flow forming offers an alternative forming strategy. As a precision plastic-forming process for axisymmetric hollow components, it involves the synchronized rotation of a tubular preform and mandrel while spinning rollers apply localized compressive loading. This induces continuous incremental plastic deformation, resulting in wall thinning and axial elongation [3,4]. Owing to its localized loading mode and incremental deformation mechanism, flow forming enables the fabrication of large-diameter thick-walled tubes at room temperature while maintaining a dense microstructure and favorable mechanical properties. In addition, the process generally requires lower forming loads and provides greater production flexibility compared with conventional hot-forming routes, thereby presenting a promising pathway for efficient, high-quality manufacturing of thick-walled tubular components.

The in-service performance of thick-walled tubes is governed by not only the geometric but also by intrinsic material characteristics and their microstructural evolution during deformation. Cu–Ni alloys are widely employed in marine systems because of their excellent resistance to seawater corrosion [5]. These alloys typically exhibit a single-phase face-centered cubic (FCC) solid-solution structure. Under room-temperature plastic deformation, microstructural evolution is dominated by dislocation multiplication, rearrangement, and substructure formation. Low-angle grain boundaries (LAGBs) progressively absorb dislocations, increasing their misorientation and gradually transforming into high-angle grain boundaries (HAGBs), thereby promoting significant grain refinement and property enhancement [6]. Such behavior is particularly pronounced in severe plastic deformation (SPD) processes. High-pressure torsion (HPT) and equal-channel angular pressing (ECAP) are two typical SPD techniques. The relationship among equivalent plastic strain (PEEQ), initial grain size ${\bar{d}}_{0}$, and refined grain size ${\bar{d}}_{t}$ reported in previous SPD cases is summarized in Table 1.

Xia et al. [11] revealed that in flow forming of a Cu–Ni tube with a thickness-to-diameter ratio of 0.1 and a total thickness reduction of 50% over three passes, the PEEQ at the outer surface reached a value as high as 23. Such deformation levels approach those typical of SPD processes, implying considerable potential for grain refinement in the outer layer of thick-walled tubes. However, unlike ECAP or HPT, where shear deformation predominates, flow forming is characterized primarily by axial tension and radial compression under an approximately plane-strain deformation state [12]. To date, the microstructural evolution of Cu–Ni alloys subjected to extreme room-temperature plane-strain deformation has not been systematically clarified. Revealing this evolution is essential for tailoring the microstructure and properties of flow-formed thick-walled Cu–Ni alloy tubes and for achieving a combined improvement in corrosion resistance and strength.

Electron backscatter diffraction (EBSD) and cellular automaton (CA) modeling constitute two complementary approaches for investigating microstructural evolution during flow forming. EBSD provides quantitative information on grain size, misorientation distribution, and grain-boundary character, offering direct experimental insight into grain refinement mechanisms. Based on EBSD, Banerjee et al. [12] demonstrated that the outer surface layer of flow-spun maraging steel tubes exhibits more pronounced refinement than the inner layer, attributed to dislocation rearrangement, subgrain formation, and progressive LAGB-to-HAGB transformation. In contrast, CA modeling allows reconstruction of dislocation accumulation, subgrain rotation, and boundary transformation under physically informed rules, thereby capturing continuous evolution pathways beyond the static snapshots provided by EBSD. Long et al. [13] employed an FEA-CA coupled framework to investigate hot flow forming of ZK61 magnesium alloy tubes, using temperature and strain fields derived from finite element model (FEM) simulations as external driving inputs for CA prediction of grain-size and dynamic recrystallization (DRX) evolution. Chen et al. [14] combined thermo-electro-mechanical FE modeling with CA simulation to analyze electrically assisted plane compression tests designed to emulate flow forming, reproducing dislocation-density accumulation and orientation evolution. However, unlike conventional flow forming of thin-walled tubes, the flow forming of thick-walled tubes is characterized not only by severe plastic deformation at the outer surface but also by a pronounced through-thickness strain gradient, which gives rise to distinct microstructural heterogeneity across the tube wall [12]. Moreover, when the dislocation-density evolution is described solely by the conventional Kocks–Mecking relation, as commonly adopted in previous studies, the role of deformation heterogeneity in promoting grain refinement may not be adequately captured.

In view of these considerations, the present study aims to systematically characterize the microstructural evolution of thick-walled Cu–Ni alloy tubes during flow forming using EBSD, with emphasis on through-thickness heterogeneity. On this basis, an FEM–CA framework is developed, in which the local strain-gradient field extracted from FEA is introduced as an external driving variable to modify the dislocation-density evolution and distribution, thereby considering the promoting effect of heterogeneous deformation on grain refinement. By integrating experimental observations with numerical predictions, this study elucidates the through-thickness microstructural evolution during flow forming of thick-walled tubes and the grain-refinement process in the outer layer, providing a mechanistic basis for microstructure control in flow-formed thick-walled Cu–Ni alloy tubes.

## 2. Materials and Methods

### 2.1. Experiments and Simulations

**Figure 1:**
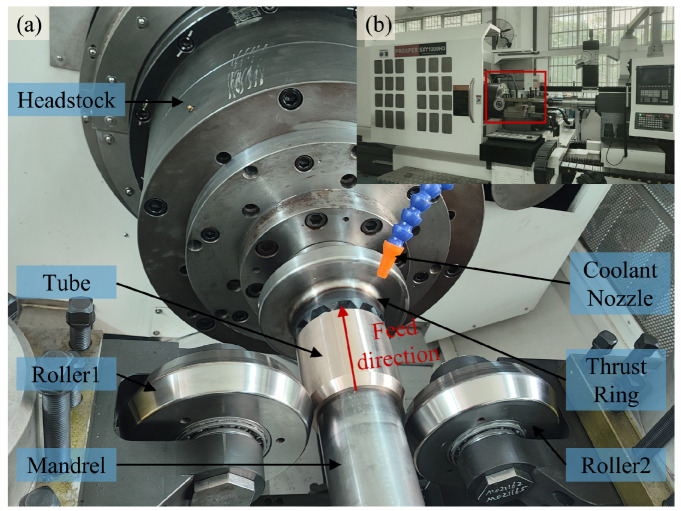
Experimental setup for two-roller reverse flow forming of thick-walled Cu–Ni alloy tubes: (**a**) close-up view of spinning platform; (**b**) overall view of spinning equipment.

A BFe10-1-1 Cu–Ni alloy tube preform was selected as the starting material in this study. The alloy primarily consists of Cu (approximately 90 wt.%) and Ni (approximately 10 wt.%). The preform had a length *l* = 120 mm, an outer diameter *D*_0_ = 128.4 mm, and an initial wall thickness *t*_0_ = 13.2, corresponding to a thickness-to-diameter ratio of 0.103. It was fabricated through vacuum casting, followed by multi-pass open-die forging and subsequent machining to achieve the required dimensions. To enhance forming efficiency, a dual-roller reverse flow-spinning configuration was employed (Figure 1a). During processing, the tail end of the tube blank was supported by a thrust ring and rotated synchronously with the spindle, while two rollers were symmetrically arranged along the circumferential direction and fed axially toward the tail end. Each roller featured a double-conical profile with a forming angle *α_p_* = 25° and a fillet radius *r_p_* = R6. The selection of process parameters was guided by Xia et al. [11]. The total thickness reduction was approximately 50%, and the detailed parameters are summarized in Table 2.

**Figure 2:**
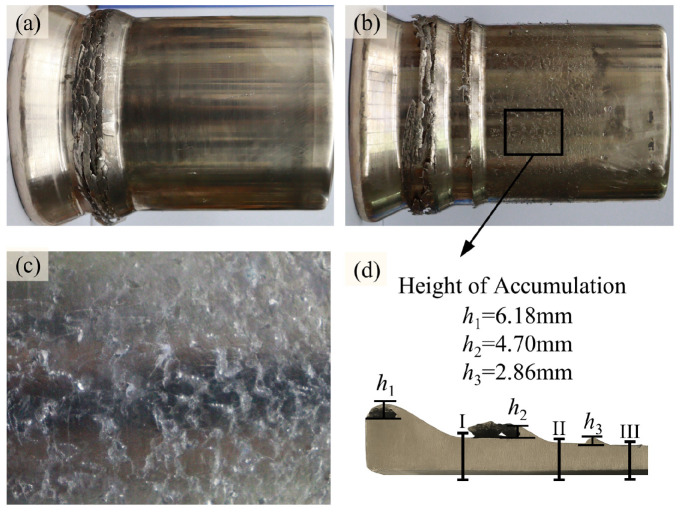
Forming results of two-roller reverse flow forming process: (**a**) tube after Pass 2; (**b**) tube after Pass 3; (**c**) fish-scale defect on outer surface; (**d**) longitudinal cross-sectional view of spun tube.

The experiments were conducted on an SXY1000HD heavy-duty spinning machine (Figure 1b), and the formed components are illustrated in Figure 2. In all three passes, noticeable material accumulation occurred on the outer surface, leading to the formation of a bulge. The bulge height—defined as the vertical distance between the bulge apex and the unformed outer surface—was measured as 6.18, 4.70, and 2.86 mm after Passes 1–3, respectively. Upon completion of the first and second passes, the tube surfaces remained smooth and free of visible defects. However, after the third pass, fish-scale-like markings appeared on the outer surface. These surface features are considered to be associated with local plastic instability, and thus indicate excessive deformation in the outer layer, and can be effectively mitigated by reducing the total thickness reduction or removed by subsequent machining [11].

**Figure 3:**
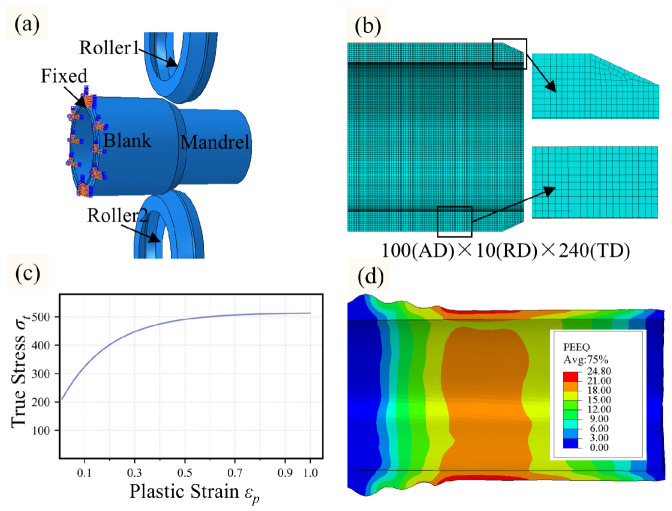
FE model for flow forming of thick-walled Cu–Ni alloy tubes: (**a**) assembly; (**b**) mesh; (**c**) stress–strain curve; (**d**) results.

To further investigate the deformation behavior and strain distribution during flow forming of thick-walled tubes, an FE model of the dual-roller reverse flow forming process was established using Abaqus/Explicit 2023 (Figure 3). The geometric configuration and processing parameters were defined to match the experimental conditions. To improve computational efficiency, several simplifications were introduced following Xia et al. [15]. A fixed constraint was applied at the tail end of the preform, while the rollers were prescribed to revolve around the tube and simultaneously feed along the axial direction (Figure 3a). The tube preform was modeled as a deformable body, whereas all tooling components—including rollers and mandrel—were treated as analytical rigid bodies. The preform was discretized using eight-node linear brick elements with reduced integration (C3D8R). The mesh consisted of 100 elements in the axial direction, 10 in the radial direction, and 240 in the circumferential direction (Figure 3b). The constitutive behavior of the material was determined from cylindrical compression tests conducted at room temperature. To ensure quasi-static testing conditions, a strain rate of 0.01 s^−1^ was selected. The experimental stress–strain data were fitted using the Voce saturation hardening model [16] in Equation (1), and the resulting flow–stress curve is depicted in Figure 3c. A penalty contact algorithm was adopted to describe the interaction between components. The friction coefficients were set to 0.05 for the roller–preform interface and 0.10 for the mandrel–preform interface, consistent with reported values [11].

Severe material accumulation is an important deformation characteristic during flow forming of thick-walled tubes and is closely associated with the formation of typical defects such as annular spallation and fish scaling [11]. Therefore, the accumulation height was selected as a key indicator for evaluating the validity of the finite element model. The simulation results are presented in Figure 3d. The predicted bulge heights after the 3 passes were 7.17, 5.05, and 3.07 mm, respectively. The mean absolute error relative to the experimental measurements was 9.19%. Moreover, after the three forming passes, the wall thicknesses predicted by the FE model were 10.97, 8.38, and 6.95 mm, respectively. The mean absolute error relative to the theoretical wall thicknesses, which were identical to the target wall thicknesses used in the experiments and are listed in Table 2, was 4.67%. This level of agreement indicates that the developed FE model can reliably capture the deformation characteristics of the process and provide accurate strain and strain-gradient histories for subsequent microstructural evolution analysis.

(1) $$\sigma_{t}=\sigma_{h}+\left(\sigma_{0}-\sigma_{h}\right)\exp\left(-m\varepsilon_{p}\right)$$

where $\sigma_{t}$ denotes the true stress; $\varepsilon_{p}$ denotes the plastic strain; $\sigma_{h}$ denotes the saturation stress (514.7 MPa); $\sigma_{0}$ denotes the initial yield stress (194.1 MPa); and *m* denotes the material constant (5.2).

### 2.2. EBSD Testing

Considering that material deformation during flow forming is primarily governed by radial compression and axial elongation, EBSD characterization was performed on longitudinal sections of the spun tubes. The sampling strategy is illustrated in Figure 2d. After completion of the third pass, longitudinal sections were obtained by wire electrical discharge machining. Along the axial direction, 3 representative regions (Section 1, Section 2 and Section 3) were selected to correspond to the microstructural states after Passes 1–3, respectively. To capture through-thickness heterogeneity, 3 sampling locations were arranged radially within each section: the outer layer (0.1 mm beneath the outer surface), the mid-layer (at the geometric center of the wall thickness), and the inner layer (0.1 mm beneath the inner surface). This design enabled systematic characterization of microstructural evolution across both forming passes and thickness positions. For the as-received preform, samples were similarly extracted from the outer surface, mid-thickness, and inner surface on the longitudinal section to serve as reference conditions.

For EBSD specimen preparation, the sectioned samples were first mechanically ground using SiC abrasive papers ranging from 220 to 3000 grit to produce a flat and uniform surface. Final surface finishing was carried out using an EMTIC3X ion-beam polishing system to ensure high surface quality suitable for reliable EBSD indexing. EBSD measurements were conducted on a JEM 2100F field-emission transmission electron microscope (Japan). A scan grid of 400 × 280 points was employed, and the magnification as well as the step size, ranging from 0.2 to 1 μm, were adjusted according to the degree of grain refinement.

The acquired EBSD datasets were post-processed using AZtecCrystal 4.0 software. Grain-boundary classification adopted 3° as the threshold for LAGBs (*θ_L_*) and 15° for HAGBs (*θ_H_*). Equivalent circular diameter was used to quantify both subgrain and grain sizes, and the aspect ratio (L/W) was determined from the best-fit ellipse of each grain. To provide a more detailed characterization of boundary evolution, a threshold of 10° was introduced to define medium-angle grain boundaries (MAGBs, *θ_M_*; Ref. [17]). Local intragranular orientation gradients were evaluated using kernel average misorientation (KAM) analysis, with a misorientation cutoff of 5°. Regions exhibiting KAM values greater than 1° were classified as high-KAM areas.

### 2.3. Construction of CA-Based Microstructure Evolution Model

Cu–Ni alloys generally exhibit a single-phase FCC solid-solution structure with relatively high stacking-fault energy. Under room-temperature plastic deformation, microstructural evolution is primarily governed by dislocation glide, cross-slip, and dynamic recovery. As deformation proceeds, dislocation substructures progressively develop, misorientation accumulates, and grain boundaries undergo gradual high-angle transformation [18]. To reproduce a CDRX-like mechanism within the CA framework, each CA iteration in the present study is decomposed into 3 principal update processes. (i) The dislocation state is updated under externally imposed deformation inputs. (ii) The evolution of grain-boundary misorientation is driven by dislocation accumulation, promoting progressive high-angle transformation. (iii) When prescribed criteria are satisfied, grain-topology splitting is triggered to represent structural refinement events.

(1) Dislocation evolution model

According to the Kocks–Mecking (K-M) framework, the evolution of dislocation density during plastic deformation can be described as the outcome of a competition between dislocation accumulation and dynamic recovery [19,20]. Within this conceptual scheme, strain hardening arises from the storage of dislocations, while recovery mechanisms reduce dislocation density through annihilation and rearrangement.

Building on this foundation, Estrin et al. [21] introduced a microstructure-based constitutive formulation that distinguishes between dislocation populations located within grains and those associated with grain boundaries. In this dual-dislocation-density framework, the average dislocation density is decomposed into 3 components: the intragranular dislocation density (*ρ_c_*), which primarily governs strain hardening; the grain-boundary statistically stored dislocation density (*ρ_ws_*), which contributes to boundary stability and impedes slip transmission; and the grain-boundary geometrically necessary dislocation (GND) density (*ρ_wg_*), which accommodates strain gradients and promotes misorientation development.

The total dislocation density at grain boundaries (*ρ_w_*) is therefore expressed as the sum of *ρ_ws_* and *ρ_wg_*:

(2) $$\rho_{w}=\rho_{ws}+\rho_{wg}$$

GNDs are intrinsically associated with non-uniform deformation [22]. The pronounced through-thickness plastic strain gradient is a typical characteristic of flow forming of thick-walled tubes, indicating significant deformation heterogeneity across the tube wall. If the evolution of grain-boundary dislocation density were described solely by conventional internal evolution equations, the gradient-induced promoting effect on grain-boundary evolution would be overlooked.

At the microscale, non-uniform deformation is mainly manifested as intergranular deformation incompatibility and the resulting local orientation gradients, which can be characterized by EBSD-based misorientation metrics such as KAM and GROD. On this basis, the GND density can be further estimated using Nye tensor analysis or proxy models (as shown in Equation (3) [14]). At the macroscopic scale, deformation heterogeneity is reflected by the spatially non-uniform distribution of the plastic strain field. For FEM simulations with sufficient mesh resolution, local plastic strain gradients and strain heterogeneity can be captured from the calculated strain field. Therefore, in this study, the heterogeneous plastic strain field extracted from FEM is introduced as an equivalent descriptor of local deformation heterogeneity and is calibrated using EBSD measurements to modify the spatial distribution of *ρ_wg_*. This treatment reflects the promoting effect of strain-gradient-induced deformation heterogeneity on grain-boundary evolution during flow forming of thick-walled Cu–Ni alloy tubes.

(3) $$\rho_{wg}\approx \frac{\theta_{\mathrm{KAM}}}{bl}$$

where *θ*_KAM_ is the kernel average misorientation expressed in radians, *b* is the Burgers vector, and *l* denotes the EBSD step size.

In FEM, deformation heterogeneity can be characterized from two perspectives. The equivalent plastic strain gradient (as shown in Equation (4), Ref. [23]) reflects spatial differences in accumulated plastic deformation. It is therefore more suitable for evaluating plastic strain accumulation and work hardening in different regions. In contrast, the Frobenius norm of the plastic strain-gradient tensor (Equations (5) and (6); Refs. [24,25]) accounts for the spatial variations in individual plastic strain components. It can more directly capture differences in local deformation modes under complex strain states. Therefore, for flow forming involving multiaxial loading and shear deformation, the Frobenius norm is more suitable for characterizing local deformation heterogeneity.

(4) $$\eta^{p}=\sqrt{{\left(\frac{\partial {\bar{\varepsilon }}^{p}}{\partial x}\right)}^{2}+{\left(\frac{\partial {\bar{\varepsilon }}^{p}}{\partial y}\right)}^{2}+{\left(\frac{\partial {\bar{\varepsilon }}^{p}}{\partial z}\right)}^{2}}$$

(5) $$\eta_{ijk}^{p}=\frac{\partial \varepsilon_{ik}^{p}}{\partial x_{j}}+\frac{\partial \varepsilon_{jk}^{p}}{\partial x_{i}}-\frac{\partial \varepsilon_{ij}^{p}}{\partial x_{k}}$$

(6) $$\eta^{p}=\frac{1}{2}\sqrt{\sum_{i}\sum_{j}\sum_{k}{\left(\eta_{ijk}^{p}\right)}^{2}},\;i,j,k\in \{a,r\}$$

where ${\bar{\varepsilon }}^{p}$ is the equivalent plastic strain and $\varepsilon_{ik}^{p}$ is the plastic strain tensor.

The extraction method for the plastic strain gradient *η^p^* based on the Frobenius norm is as follows: For a node x_0_ in the FEM, a neighborhood node set *N_r_* within a radius *R* is first identified (Equation (7)). A distance-decaying weighting function is then introduced, and a weighted least-squares scheme is employed to estimate the spatial gradients of the plastic strain components on the longitudinal section (Equations (8) and (9)) [26]. Based on these gradient components, the plastic strain-gradient tensor is constructed, and its Frobenius norm is calculated to obtain the scalar strain-gradient measure *η^p^* (Equations (7) and (8)). To ensure that the neighborhood spans at least 3 layers of elements in the thickness direction—while still retaining sufficient sensitivity to capture the sharp strain-gradient variations (*η^p^*) near the outermost surface of the tube preform—the neighborhood radius was set to *R* = 3 mm.

(7) $$N_{R}(x_{0})=\left\{n|\left\|x_{n}-x_{0}\right\|\leq R\right\},\;x\in {\mathbb{R}}^{2}$$

(8) $$\omega_{n}={\left(1-{\left(\frac{\left\|x_{n}-x_{0}\right\|}{R}\right)}^{2}\right)}^{2},\;n\in N_{R}(x_{0})$$

(9) $$\left[\begin{array}{c} \varepsilon_{ij,a}^{p}(x_{0})\\ \varepsilon_{ij,r}^{p}(x_{0}) \end{array}\right]={\left(\sum_{n\in N_{R}(x_{0})}\omega_{n}(x_{n}-x_{0}){\left(x_{n}-x_{0}\right)}^{T}\right)}^{-1}\left(\sum_{n\in N_{R}(x_{0})}\omega_{n}(x_{n}-x_{0})\left(\varepsilon_{ij}^{p}(x_{n})-\varepsilon_{ij}^{p}(x_{0})\right)\right)$$

**Figure 4:**
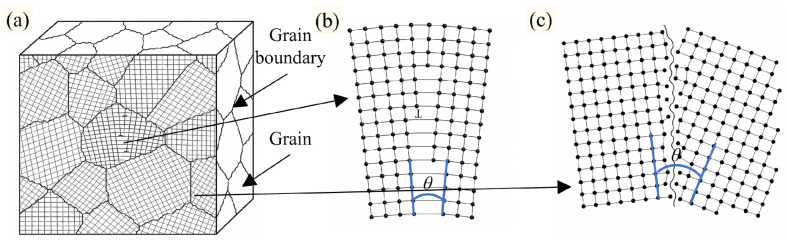
Schematic illustration of grain boundaries: (**a**) grain; (**b**) LAGB; (**c**) HAGB.

LAGBs (*θ* < 15°, Figure 4b) can be described as ordered dislocation-array structures. Within this regime, *ρ_wg_* can be reasonably approximated as being proportional to the boundary misorientation angle *θ* [27]. However, once the boundary evolves beyond the low-angle regime (*θ* > 15°, Figure 4c), the dislocation core fields begin to overlap, and the boundary structure progressively loses its dependence on discrete dislocation-array characteristics. Under these conditions, dislocation-density characterization approaches based on the assumption of non-interacting dislocation arrays are no longer applicable [12]. Accordingly, in the present model, the contribution of *ρ_wg_* is truncated for HAGBs. After incorporating a misorientation-dependent weighting function (Equations (9) and (10)), the relationship between *ρ_wg_* and the plastic strain gradient is expressed in Equation (11); Ref. [28].

(10) $$w_{\mathrm{gnd}}(\theta )=\mathrm{clip}\left(\frac{\theta }{\theta_{H}},0,1\right)$$

(11) $${\tilde{w}}_{\mathrm{gnd}}(\theta )=\frac{w_{\mathrm{gnd}}(\theta )}{\sum_{j\in gb}f(\theta_{j})w_{\mathrm{gnd}}(\theta_{j})}$$

(12) $$\rho_{wg}(\theta )={\tilde{w}}_{\mathrm{gnd}}(\theta )\frac{c_{g}\eta^{p}}{b}$$

where *c_g_* denotes the geometric correction factor that incorporates the effects of crystallographic orientation distribution, FE mesh density, and the differences between the grain boundary and *ρ_c_*. This parameter was calibrated against EBSD measurements, and its optimized value was 4.29 × 10^3^ (calibration procedure in Appendix A); and *f*(*θ*) denotes the fraction of grain boundaries with misorientation angle *θ*, which is a CA state variable.

Since *ρ_c_* and *ρ_ws_* do not explicitly enter the iterative calculations for misorientation evolution or grain-size updating, their governing equations are not elaborated here. For completeness, the corresponding update formulations are provided in Appendix B.

(2) Grain-boundary misorientation evolution model

Under a CDRX-like mechanism, subgrain boundaries progressively absorb dislocations and undergo continuous lattice rotation, leading to the accumulation of misorientation. When the misorientation angle of a subgrain reaches a critical threshold, the subgrain transforms into an independent grain, thereby contributing to grain refinement [29]. The rotation rate of subgrains is influenced by factors such as subgrain size and local dislocation density [30]. Since the spinning process in the present study is conducted at room temperature, the thermal effects can be considered approximately constant. Under such conditions, orientation evolution exhibits limited sensitivity to strain rate [31]. Therefore, the temperature- and strain-rate-dependent coefficients in the original formulation are consolidated into effective material constants. This simplification leads to an incremental update expression for the misorientation angle, expressed in the following form:

(13) $$\frac{d\theta }{d{\bar{\varepsilon }}^{p}}=K_{\theta }b\sqrt{\rho_{wg}}{\left(\frac{{\bar{d}}_{0}}{{\bar{d}}_{t}}\right)}^{1/3}{\left(E_{\theta }\right)}^{m_{1}}$$

(14) $$E_{\theta }=b^{2}\left[\rho_{wg}\left(1-\ln\frac{10b}{\sqrt{\rho_{wg}}}\right)+\frac{2\theta }{{\bar{d}}_{t}^{s}b}\left(1+\ln\frac{\theta_{H}}{\theta }\right)\right]$$

where K_θ_ denotes the effective material coefficient governing the rate of misorientation evolution and was calibrated based on EBSD measurements; *m*_1_ denotes the sensitivity exponent describing the dependence of subgrain rotation rate on stored energy (0.1; [12]); ${\bar{d}}_{0}$ denotes the initial mean grain size, which is a CA state variable; ${\bar{d}}_{t}$ denotes the current mean grain size, which is a CA state variable; and ${\bar{d}}_{t}^{s}$ denotes the current mean subgrain size, which is a CA state variable.

(3) Grain-splitting model

In previous studies, individual refinement events have often been idealized using a fixed number of subdivisions. Kim & Chang [32] suggested that the dihedral angles at stable triple junctions tend to approach 120° and accordingly approximated a grain-refinement event as an “average three-way division” (*n*_min_ = 3). In contrast, Tóth et al. [33] considered lattice distortion induced by grain-boundary constraints and pointed out that deformation compatibility differs between the grain interior and the near-boundary region. This heterogeneity favors the formation of finer substructures, and a representative case may be approximated by subdivision into nine subgrains (*n*_max_ = 9). However, subgrain formation during actual plastic deformation is inherently stochastic rather than deterministic. Larger grains typically exhibit more pronounced internal strain heterogeneity and stronger orientation gradients, which increase the likelihood of forming multiple subgrain regions [34]. Conversely, as grain size decreases, the capacity for further subdivision diminishes, making refinement progressively more difficult [35]. Taking these physical considerations into account, the present work introduces a modified formulation for the number of grain subdivisions (Equations (14) and (15)), while the probability of a splitting event is described by Equation (16):

(15) $$p_{n}(n)=\frac{\exp(w_{n})}{\sum_{k=3}^{9}\exp(w_{k})}$$

(16) $$p_{s}(n)=c_{2}{\left(\frac{d_{t}}{{\bar{d}}_{0}}\right)}^{2}$$

(17) $$w_{n}=c_{1}\ln n\left(\frac{d_{t}}{{\bar{d}}_{0}}-1\right)$$

where *c*_1_ denotes the scaling coefficient that regulates the dispersion of the subgrain-number distribution, thereby controlling the statistical variability of subdivision events. This parameter was calibrated based on EBSD measurements. *c*_2_ denotes the scaling coefficient that governs the ease of grain splitting, reflecting the sensitivity of subdivision probability to the local microstructural state; it was likewise determined through EBSD-based calibration.

In the flow forming of thick-walled tubes, the pronounced through-thickness strain gradient intensifies deformation incompatibility across adjacent regions. This enhanced heterogeneity promotes the development of intragranular orientation gradients and accelerates grain refinement. If *θ_L_* were retained as the criterion for subdivision, the refinement rate would be underestimated under such strong strain-gradient conditions. Moreover, MAGBs can be regarded as a transitional regime in which subgrain boundaries evolve from low-angle dislocation-array structures toward more mature and continuous grain-boundary configurations [17]. Based on this physical interpretation, the present model adopts the *θ_M_* as the criterion for permitting grain splitting. Specifically, when *θ* > *θ_M_*, the corresponding subgrain boundary is incorporated into the splittable set during subsequent CA iterations. The expected misorientation angle at the moment of splitting is regulated by *c*_2_.

In the present CA framework, grain-shape deformation is treated as a module relatively independent of grain refinement. Although including this process could simulate elongated grains, it would greatly increase computational cost and reduce numerical stability. Since this work mainly focuses on the statistical evolution of average grain size, only grain-refinement-related processes are considered, and grain-shape deformation is not explicitly introduced.

(4) Calibration of CA model parameters

Since the CA model targeted ultrafine-grain formation in the outer surface layer, its parameters were calibrated using EBSD data from that layer. The calibration procedure was carried out in two stages. Initially, a trial-and-error approach was employed to preliminarily constrain the feasible ranges of key parameters, ensuring that the simulated results were consistent with experimental observations in both magnitude and overall evolution trend. This step provided a physically reasonable parameter space for subsequent optimization. Following this pre-screening, a Bayesian optimization algorithm was introduced to identify the optimal parameter set within the defined search domain. Compared with conventional manual fitting, Bayesian optimization improves efficiency and avoids convergence toward local minima by systematically exploring the parameter space.

In EBSD analysis, certain LAGBs that do not form fully enclosed subgrain structures may disappear during subsequent rearrangement. As a result, the measured LAGB fraction can be higher than the actual proportion of subgrain boundaries that effectively delineate subgrains [36]. To mitigate the influence of this uncertainty, the objective function for parameter calibration was defined by minimizing the errors in the average subgrain size and average grain size. The simulated trends of the LAGB and MAGB fractions were required to be qualitatively consistent with the EBSD results, rather than serving as strict quantitative constraints. The optimized parameter ranges and the final selected values are outlined in Table 3.

## 3. Results and Discussion

### 3.1. Pass-Dependent and Through-Thickness Microstructural Evolution

**Figure 5:**
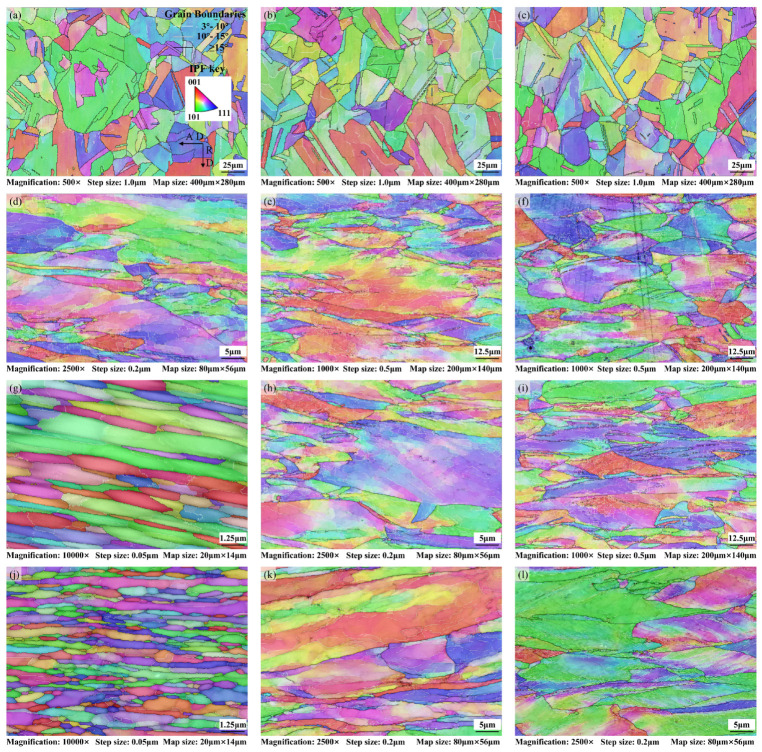
EBSD results: (**a**) outer surface of the preform; (**b**) mid-thickness of the preform; (**c**) inner surface of the preform; (**d**) outer surface after Pass 1; (**e**) mid-thickness after Pass 1; (**f**) inner surface after Pass 1; (**g**) outer surface after Pass 2; (**h**) mid-thickness after Pass 2; (**i**) inner surface after Pass 2; (**j**) outer surface after Pass 3; (**k**) mid-thickness after Pass 3; (**l**) inner surface after Pass 3.

The microstructural morphologies, grain-size statistics, grain-boundary distributions, and KAM results obtained from EBSD are presented in Figure 5, Table 4 and Table 5.

In the preform (Figure 5a–c), the microstructure at the outer, middle, and inner layers is characterized predominantly by coarse equiaxed grains, within which annealing twins are frequently observed. After excluding Σ3 twin boundaries, the average grain size and average subgrain size are both approximately 20 μm, with negligible variation along the wall thickness. This confirms that the initial material exhibits a relatively homogeneous annealed microstructure typical of Cu–Ni alloys [37].

After Pass 1 (Figure 5d–f), the annealing twins are markedly reduced. The initially equiaxed grains become elongated along the axial direction, forming a banded morphology. The mean aspect ratio (L/W) at all three thickness layers reaches approximately 5.35, reflecting the pronounced morphological fibering induced by radial compression and axial stretching during flow forming. Concurrently, a dense network of LAGBs develops within the grains, with the average LAGB fraction increasing from 20.40 to 64.73%. The increase in mean KAM value and the substantial expansion of high-KAM regions (HA), together with the fragmented subgrain boundaries, indicate rapid accumulation of dislocations during Pass 1. Under dynamic recovery-dominated rearrangement, dislocations progressively organize into dislocation walls, leading to the formation and gradual interconnection of numerous low-angle subgrain boundaries [38]. Meanwhile, the MAGB fraction rises from 0.63 to 10.96%, suggesting that continued subgrain rotation and coalescence promote further misorientation buildup and drive grain-boundary evolution toward higher angles. As a result, a clear distinction emerges between subgrain size and grain size, and a through-thickness gradient in refinement becomes evident. The outer surface exhibits the most pronounced refinement, with the average subgrain size and grain size decreasing from approximately 20 to 1.85 and 6.74 μm, respectively.

Following Pass 2 (Figure 5g–i), the microstructural evolution becomes distinctly asynchronous along the thickness direction. At the outer surface, the HAGB fraction increases sharply from 22.8 to 66.4%, and the average grain size is refined to the submicron scale (0.8 μm). Simultaneously, the mean KAM value decreases from 1.12° to 0.33°, and the area fraction of high-KAM regions drops from 46.93 to 3.37%. Grain boundaries appear continuous and well defined. These changes indicate that subgrain rotation and misorientation accumulation are significantly accelerated in the outer layer, promoting the development of a continuous HAGB network. The marked reduction in intragranular orientation gradients further suggests a transition in the dominant mechanism from rapid substructure generation and accumulation toward HAGB-mediated ultrafine-grain formation [39,40]. In contrast, the grain sizes in the mid-thickness and inner regions remain 8.2 and 10.94 μm, respectively, showing no significant reduction compared with the end of Pass 1. Their KAM values remain relatively high, indicating that microstructural evolution in these regions is still dominated by substructure accumulation and progressive misorientation increase.

After Pass 3 (Figure 5j–l), further grain refinement occurs at the outer surface, but the magnitude of change decreases noticeably. The HAGB fraction increases to 82.2%, while the mean KAM remains low (0.29°), and high-KAM regions account for only 2.51% of the area. Meanwhile, both LAGB and MAGB fractions continue to decline, suggesting that the formation rate of new subgrain boundaries diminishes and that existing boundaries increasingly evolve toward higher misorientation states. These observations indicate that microstructural evolution at the outer layer has entered a saturation regime. Morphologically, the banded structure is retained, with a mean aspect ratio of approximately 5.47. By comparison, the mid-layer and inner-layer microstructures remain in a delayed evolution state, still characterized primarily by substructure accumulation and gradual high-angle development.

### 3.2. Formation Pathway of Ultrafine Grains

**Figure 6:**
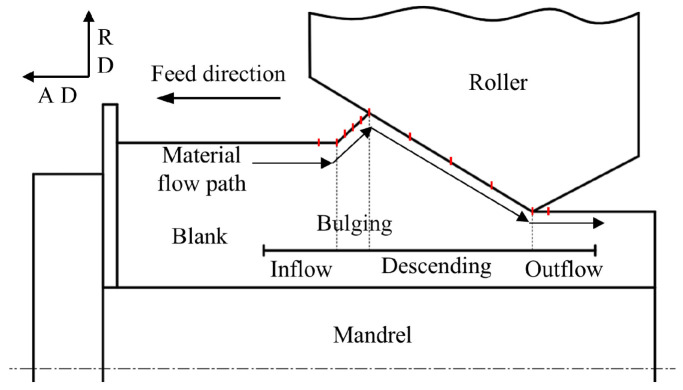
Schematic of material flow path and sampling locations.

**Figure 7:**
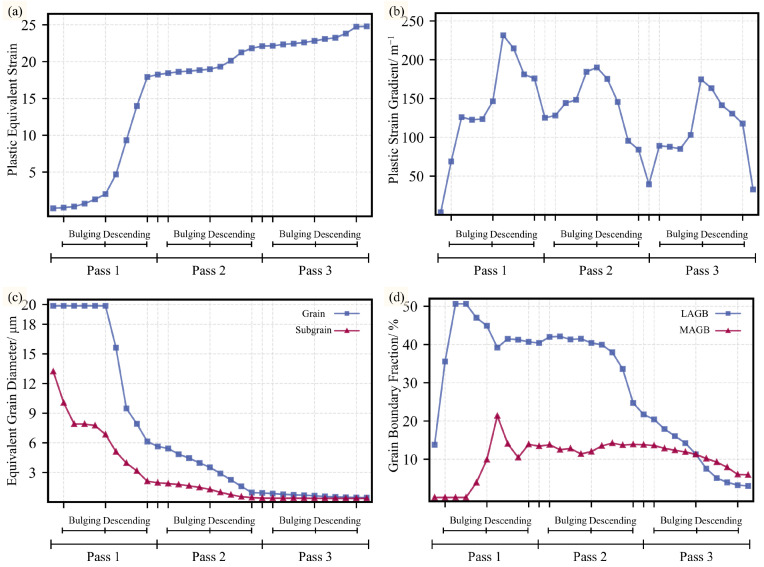
CA inputs and results: (**a**) PEEQ; (**b**) strain gradient; (**c**) grain size; (**d**) grain-boundary fractions.

Based on the material flow characteristics during flow forming, the deformation path can be divided into four sequential stages: the inflow zone, bulging zone, thinning zone, and outflow zone (Figure 6). To characterize the microstructural evolution of the outer surface layer throughout these stages, a representative outer surface node was selected from the FE model. The evolution histories of PEEQ and plastic strain gradient at this node were extracted and used as inputs for the CA simulation. Simultaneously, the CA-predicted grain size and grain-boundary fractions at different deformation stages were analyzed to describe the corresponding microstructural states. The results are depicted in Figure 7, while representative grain morphologies are provided in Appendix C.

For the three passes, the EBSD-measured average subgrain sizes were 1.85, 0.54, and 0.33 μm, respectively, while the corresponding predictions of the cross-scale grain refinement model were 1.96, 0.45, and 0.37 μm. The corresponding errors were 5.95%, 16.67%, and 12.12%, with an average error of 11.58%. In addition, the EBSD-measured average grain sizes after the three passes were 6.74, 0.80, and 0.39 μm, respectively, while the model predictions were 5.63, 0.92, and 0.45 μm. The corresponding errors were 16.47%, 15.00%, and 15.38%, with an average error of 15.62%. Over the refinement process from coarse grains (19.14 μm) to ultrafine grains (0.33 μm), the CA framework successfully captures the overall evolution trend of microstructure during flow forming of thick-walled tubes, indicating its reliability in reproducing stage-dependent refinement behavior.

In Figure 7a, PEEQ increases progressively over the three passes, with the most pronounced increments occurring in the bulging and thinning zones of each pass, particularly during Pass 1. These two zones therefore constitute the primary plastic deformation regions for the outer surface layer. In Figure 7b, the plastic strain gradient remains at a relatively high level in both the bulging and thinning zones and exhibits stage-wise fluctuations. This behavior reflects substantial strain heterogeneity and deformation incompatibility between neighboring grains within the active deformation region. Consequently, the outer layer experiences a cumulative loading history characterized by the simultaneous presence of “high strain accumulation and high strain gradient”, providing sustained driving forces for substructure formation, misorientation accumulation, and progressive grain-boundary high-angle development.

The evolution of grain and subgrain sizes (Figure 7c) exhibits a distinct stage-dependent response. During the bulging zone of Pass 1, subgrain refinement occurs rapidly, while the average grain size remains nearly unchanged, indicating that deformation is primarily accommodated by dislocation organization and rapid substructure formation. Upon entering the thinning zone, a pronounced decrease in grain size is observed, suggesting that grain-boundary high-angle transformation is activated in this stage. In Pass 2, further refinement occurs, and grain size in the thinning zone decreases to the submicron range. By Pass 3, refinement continues but at a markedly reduced rate. The size gap between grains and subgrains narrows significantly and tends toward convergence, indicating that the refinement process approaches a plateau and the microstructure enters a near-saturated state [35].

The temporal evolution of grain-boundary fractions (Figure 7d) further clarifies the underlying mechanism transitions and is qualitatively consistent with the EBSD-observed grain-boundary evolution. In the bulging zone of Pass 1, the LAGB fraction rises rapidly while the MAGB fraction remains negligible, consistent with a substructure-dominated stage. During the thinning zone of Pass 1, the MAGB fraction increases substantially, whereas the LAGB fraction decreases and stabilizes at approximately 40%, indicating active misorientation accumulation and high-angle transformation that drive substantive grain-scale refinement. In Pass 2, the LAGB and MAGB fractions fluctuate within ranges of approximately 40% and 12–14%, respectively, reflecting a dynamic balance between subgrain-boundary generation and misorientation growth. In Pass 3, both fractions exhibit an overall decline, suggesting that the formation of new subgrain boundaries becomes less frequent, while existing boundaries continue migrating toward higher misorientation states. This behavior marks the transition into a mature plateau regime of microstructural evolution.

## 4. Conclusions

(1) The microstructural evolution of thick-walled Cu–Ni alloy tubes during flow forming follows a CDRX-like continuous high-angle boundary development pathway. Owing to the pronounced through-thickness strain gradient, the outer layer undergoes accelerated refinement and forms a stable banded ultrafine-grained structure, whereas the mid-thickness and inner layers show delayed refinement dominated by substructure accumulation and gradual misorientation increase.

(2) The coupled FEA-CA framework successfully reproduced the outer-layer grain refinement from 19.14 μm to 0.33 μm during multi-pass flow forming, with mean absolute errors of 15.67% for grain size and 14.30% for subgrain size, thereby clarifying the underlying multiscale grain-refinement mechanism during thick-walled tube flow forming.

(3) Ultrafine grains in the outer layer form through progressive refinement of coarse grains, and the governing mechanism is strongly pass dependent. Pass 1 is characterized by rapid substructure establishment, Pass 2 by the most efficient grain refinement, and Pass 3 by limited new boundary formation and gradual evolution toward a saturated microstructure.

The limitations and future work are detailed as follows:

(1) In the present study, the Frobenius norm of the plastic strain-gradient tensor was adopted to characterize macroscopic deformation heterogeneity. However, a systematic comparison with other strain-gradient measures was not conducted. In future work, different methods for calculating *η^p^*, such as the gradient of equivalent plastic strain, directional strain gradients, and other tensor-norm-based descriptors, will be compared to further assess their influence on CA-based microstructure prediction.

## Figures and Tables

**Table 1:** Comparison of equivalent plastic strain and grain refinement in Cu and Cu alloys processed by SPD.

	Process	Material	${\bar{d}}_{0}$/μm	PEEQ	${\bar{d}}_{t}$/μm
Edalati et al. [7]	HPT	Cu	150	7.6	0.3
Khereddine et al. [8]	HPT	Cu-2.5Ni-0.6Si	20	28	0.2
Higuera-Cobos and Cabrera [9]	ECAP	Cu	5.5	13.2	0.5
Hadj Larbi et al. [10]	ECAP	Cu-2.5Ni-0.6Si	20	6	0.9

**Table 2:** Process parameters for two-roller reverse flow forming of BFe10-1-1 thick-walled tube.

Pass	Initial Wall Thickness/mm	Thinning Ratio/%	Feed Rate/(mm/r)
1	13.2	21.9	0.2
2	10.3	22.3	0.15
3	8	17.5	0.15

**Table 3:** Parameter ranges and optimal values from Bayesian optimization.

	Bayesian Optimization Search Ranges		Optimal Value
	Minimum	Maximum	
*K_θ_*	100	500	280
*c*_1_	1	10	8.5
*c*_2_	0.01	0.5	0.15

**Table 4:** Summary of grain-size statistics at different passes and thickness positions.

		Subgrains		Grains		
		Mean Size/μm	Std/μm	Mean Size/μm	Std/μm	L/W
Blank	Outer layer	18.77	14.57	19.55	15.2	3.54
	Mid-layer	20.4	15.55	21.83	16.18	3.37
	Inner layer	18.26	15.44	18.69	16.14	3.6
	Mean	19.14	15.19	20.02	15.84	3.50
Pass 1	Outer layer	1.85	2.2	6.74	10.55	5.56
	Mid-layer	4.6	5.18	11.66	18.24	5.62
	Inner layer	5.67	7.35	11.56	13.9	4.87
	Mean	4.04	4.91	9.98	14.23	5.35
Pass 2	Outer layer	0.54	0.39	0.8	0.68	6.56
	Mid-layer	2.7	3.99	8.2	10.8	5.09
	Inner layer	4.89	5.38	10.94	14.01	6.24
	Mean	2.71	3.25	6.65	8.50	5.96
Pass 3	Outer layer	0.33	0.22	0.39	0.34	5.47
	Mid-layer	1.54	2.35	3.87	7.45	6.71
	Inner layer	2.92	4.67	8.51	10.38	5.19
	Mean	1.60	2.41	4.26	6.06	5.79

**Table 5:** Summary of grain-boundary fractions and KAM statistics.

		Grain Boundaries			KAM	
		LAGB/%	MAGB/%	HAGB/%	Mean Value/°	HA/%
Blank	Outer layer	12.9	0.84	86.2	0.96	37.22
	Mid-layer	28.6	0.18	71.2	0.54	15.95
	Inner layer	19.7	0.86	79.4	0.71	21.31
	Mean	20.40	0.63	78.93	0.74	24.83
Pass 1	Outer layer	62.8	14.4	22.8	1.12	46.93
	Mid-layer	66.2	11.1	22.6	1.53	71.69
	Inner layer	65.2	7.38	27.4	1.38	65.24
	Mean	64.73	10.96	24.27	1.34	61.29
Pass 2	Outer layer	22.3	11.3	66.4	0.33	3.37
	Mid-layer	67.2	9.23	23.6	1.17	50.84
	Inner layer	61.8	8.79	29.5	1.55	72
	Mean	50.43	9.77	39.83	1.02	42.07
Pass 3	Outer layer	12.1	5.69	82.2	0.29	2.51
	Mid-layer	63	10.9	26.1	0.73	24.45
	Inner layer	71.8	5.16	23.1	1.32	62.89
	Mean	48.97	7.25	43.80	0.78	29.95

**Figure A1:**
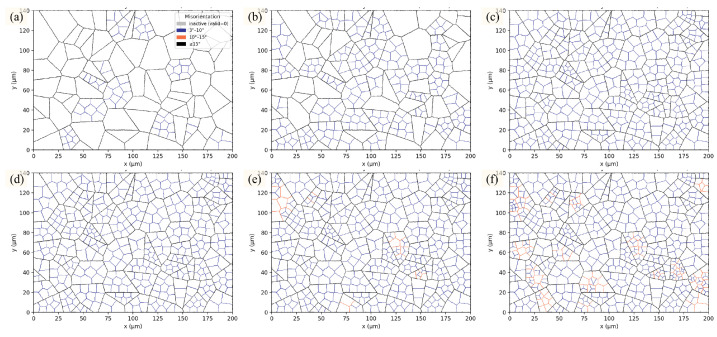

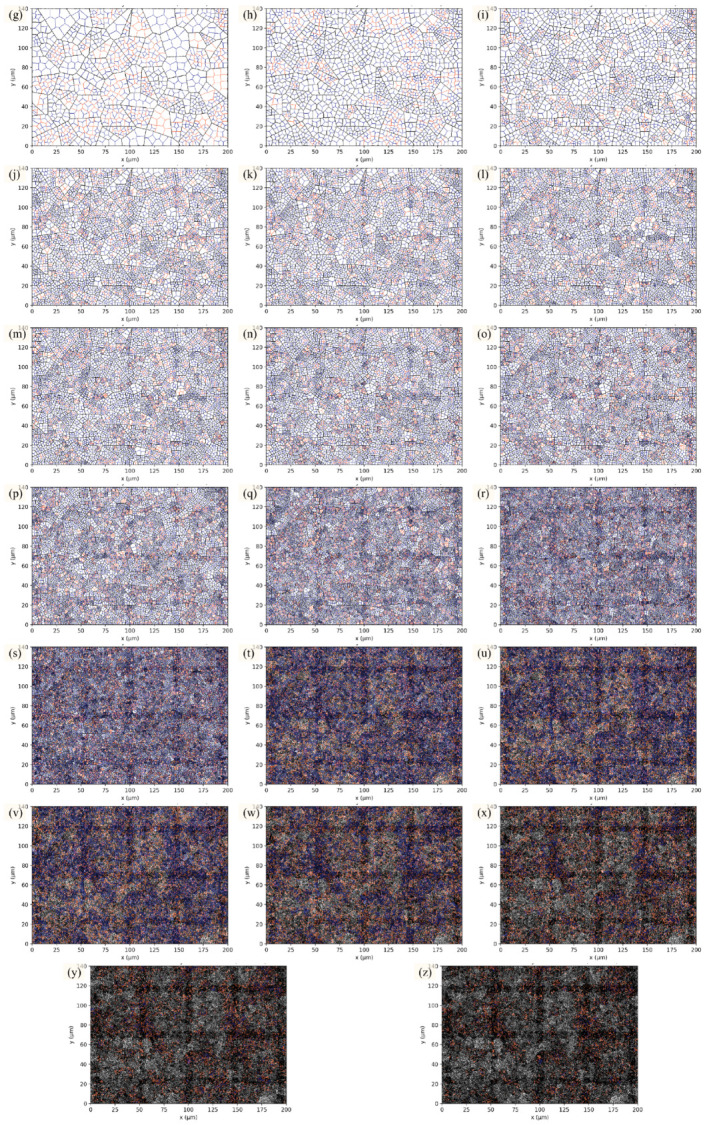
CA-predicted microstructural evolution during multi-pass flow forming: (**a**–**k**) Pass 1; (**l**–**u**) Pass 2; (**v**–**z**) Pass 3.

## Data Availability

The original contributions presented in this study are included in the article. Further inquiries can be directed to the corresponding author.

## Appendix A

The *η^p^* values at the outer surface obtained from FEA after Passes 1–3 (Figure 5d,g,i) are 126, 39, and 31 m^−1^, respectively.

Following the widely adopted approach of estimating dislocation density from KAM, the local lattice curvature can be characterized by the average misorientation angle (*θ*_KAM_) between each pixel and its surrounding 3 × 3 neighborhood. A proxy measure of GND density can be obtained through Equation (3):

The *ρ_wg_* corresponding to different misorientation-angle intervals is summarized in Table 5. The overall evolution trend is in good agreement with the assumptions adopted in the CA model. Specifically, within the range of 3° ≤ *θ* ≤ 15°, *ρ_wg_* exhibits an approximately linear increase with increasing misorientation angle, a trend that is particularly pronounced after Pass 1. Once *θ* > 15°, *ρ_wg_* decreases markedly, indicating that the grain-boundary structure approaches a mature high-angle configuration in which the discrete dislocation-array description becomes less applicable.

Using Equations (9)–(11) to calculate *ρ_wg_* and assuming a uniform grain-boundary fraction *f* across different misorientation intervals, the initial estimate of *c_g_* obtained by the least-squares method was 5.35 × 10^3^. Based on this preliminary value, CA iterations were performed and the resulting *ρ_wg_* distribution was compared with the experimental data in Table 5. After calibration, the optimized value of *c_g_* was 4.29 × 10^3^.

**Table A1:** Distribution of grain-boundary GND density.

*θ*/°		*ρ_wg_*/m^−2^		
Min	Max	Pass 1	Pass 2	Pass 3
3	4	6.03 × 10^14^	3.31 × 10^14^	2.90 × 10^14^
4	5	7.25 × 10^14^	3.63 × 10^14^	2.59 × 10^14^
5	6	9.00 × 10^14^	3.10 × 10^14^	2.20 × 10^14^
6	7	1.07 × 10^15^	4.28 × 10^14^	2.95 × 10^14^
7	8	1.23 × 10^15^	4.69 × 10^14^	4.31 × 10^14^
8	9	1.37 × 10^15^	4.10 × 10^14^	4.37 × 10^14^
9	10	1.56 × 10^15^	4.51 × 10^14^	4.45 × 10^14^
10	11	1.62 × 10^15^	5.62 × 10^14^	5.12 × 10^14^
11	12	1.88 × 10^15^	5.22 × 10^14^	5.67 × 10^14^
12	13	2.13 × 10^15^	5.56 × 10^14^	5.16 × 10^14^
13	14	2.08 × 10^15^	6.53 × 10^14^	5.91 × 10^14^
14	15	2.28 × 10^15^	6.40 × 10^14^	6.31 × 10^14^
15	16	9.46 × 10^14^	7.66 × 10^13^	1.11 × 10^14^
16	17	5.81 × 10^14^	9.18 × 10^13^	9.69 × 10^13^
17	18	6.44 × 10^14^	1.12 × 10^14^	8.24 × 10^13^
18	19	7.17 × 10^14^	9.39 × 10^13^	9.29 × 10^13^
19	20	6.15 × 10^14^	9.58 × 10^13^	9.81 × 10^13^

## Appendix B

After incorporating the externally imposed strain-gradient correction into the distribution of *ρ_wg_*, the evolution equations for *ρ_c_* and *ρ_ws_* are expressed in Equations (A1)–(A3) [21]. These equations retain the fundamental structure of the K-M model, in which the first term represents dislocation accumulation due to plastic deformation and the second term accounts for dynamic recovery through dislocation annihilation and rearrangement. On this basis, an additional third term is introduced to describe dislocation migration.

(A1) $$\frac{d\rho_{c}}{d{\bar{\varepsilon }}^{p}}=k_{1}\sqrt{\rho_{c}}-k_{2}\rho_{c}-\frac{\beta_{cw}}{b{\bar{d}}_{t}{\left(1-f\right)}^{1/3}}\rho_{c}$$

(A2) $$\frac{d\rho_{ws}}{d{\bar{\varepsilon }}^{p}}={\dot{\rho }}_{c}r_{w}k_{1}\sqrt{\rho_{ws}+\rho_{wg}}-r_{w}k_{2}\rho_{ws}+\frac{\beta_{cw}{\left(1-f\right)}^{2/3}}{b{\bar{d}}_{t}f}\rho_{c}$$

(A3) $$k_{1}=\frac{2m\sigma_{\mathrm{sat}}}{M\alpha \mu b},\;k_{2}=2m$$

where *a_w_* and *r_w_* denote the grain-boundary correction coefficients that account for grain-boundary pinning and dislocation sink effects, respectively. For Cu–Ni alloys at room temperature, their empirical values are 1.2 and 0.9. *d_t_* denotes the equivalent grain diameter, which is a CA state variable; *f* denotes the grain-boundary fraction, which is a CA state variable; *M* denotes the Taylor factor (3.0); *α* denotes the dislocation interaction constant (0.35); *b* denotes the Burgers vector magnitude (0.25 nm); *μ* denotes the shear modulus (55 GPa); and *m* denotes the strain-hardening parameter, which is determined from the derivative of the corresponding stress–strain curve.

## References

[1] Zhou L., Gong S., Yuan L., Wang X., Wang Z. (2025). Evaluation on collapse behaviour of thick-walled pipes with corrosion defect under combined external pressure, axial tension and bending moment. Structures.

[2] Jia L., Li Y., Hui T., Zhang Y. (2019). Numerical Simulation and Experimental Research on Microstructural Evolution During Compact Hot Extrusion of Heavy Caliber Thick-Wall Pipe. Chin. J. Mech. Eng..

[3] Xia Q., Xiao G., Long H., Cheng X., Sheng X. (2014). A review of process advancement of novel metal spinning. Int. J. Mach. Tools Manuf..

[4] Xia Q., Yuan S., Xiao G., Long J., Cheng X. (2021). Meso-modelling study of the mechanical response and texture evolution of magnesium alloy during hot compression. Mater. Today Commun..

[5] Zheng Z.Q., Lu J.C., Wang Z.B., Zhao Y., He B., Zheng Y.G. (2025). Improving corrosion resistance of copper-nickel alloys by the microalloying-facilitated formation of protective corrosion product films. Corros. Sci..

[6] Li X., Lu K. (2021). Refining grains of metals through plastic deformation: Toward grain size limits. Acc. Mater. Res..

[7] Edalati K., Fujioka T., Horita Z. (2008). Microstructure and mechanical properties of pure cu processed by high-pressure torsion. Mater. Sci. Eng. A.

[8] Khereddine A.Y., Larbi F.H., Kawasaki M., Baudin T., Bradai D., Langdon T.G. (2013). An examination of microstructural evolution in a cu–ni–si alloy processed by HPT and ECAP. Mater. Sci. Eng. A.

[9] Higuera-Cobos O.F., Cabrera J.M. (2013). Mechanical, microstructural and electrical evolution of commercially pure copper processed by equal channel angular extrusion. Mater. Sci. Eng. A.

[10] Hadj Larbi F., Azzeddine H., Baudin T., Mathon M.-H., Brisset F., Helbert A.-L., Kawasaki M., Bradai D., Langdon T.G. (2015). Microstructure and texture evolution in a cu–ni–si alloy processed by equal-channel angular pressing. J. Alloys Compd..

[11] Xia Q., Zhao J., Xiao G., Tang D., Cui H. (2026). A CNN-LSTM model for strain-geometry state prediction and defect control in multi-pass flow forming of thick-walled tubes. J. Intell. Manuf..

[12] Banerjee A., Nelson K., Milliken D., Da Silva L. (2025). Sustainable manufacturing of maraging steel seamless tube via flow forming: Structure–property relations. Arch. Civ. Mech. Eng..

[13] Long J., Xiao G., Xia Q., Wang X. (2024). Study of microstructure evolution of magnesium alloy cylindrical part with longitudinal inner ribs during hot flow forming by coupling ANN-modified CA and FEA. J. Magnes. Alloys.

[14] Chen C., Xia Q., Zhou H., Zhao J., Qin Y., Xiao G. (2024). Study of the microstructure evolution of alloy structural steel and inhomogeneity effect of the microscale pulsed currents during current-assisted plane strain compressions by modeling a novel cellular automata method. Mater. Charact..

[15] Xia Q., Long J., Xiao G., Yuan S., Qin Y. (2021). Deformation mechanism of ZK61 magnesium alloy cylindrical parts with longitudinal inner ribs during hot backward flow forming. J. Mater. Process. Technol..

[16] Pokharel R., Niu T., Ricci S., Clausen B., Balogh L., Ravkov L., Martinez R., Lee C., Vogel S., Cady C.M. (2024). Alloying effects on deformation induced microstructure evolution in copper. Sci. Rep..

[17] Cheng W., Bai Y., Ma S., Wang L., Wang H., Yu H. (2019). Hot deformation behavior and workability characteristic of a fine-grained mg-8Sn-2Zn-2Al alloy with processing map. J. Mater. Sci. Technol..

[18] Devi Janani R., Salman S.A., Pavithra Priyadharshini K., Karthik V. (2021). Effect of composition on the stacking fault energy of copper-nickel alloys using molecular dynamics simulations. Mater. Today Proc..

[19] Kocks U.F., Mecking H. (2003). Physics and phenomenology of strain hardening: The FCC case. Prog. Mater. Sci..

[20] Mecking H., Kocks U.F. (1981). Kinetics of flow and strain-hardening. Acta Metall..

[21] Estrin Y., Tóth L.S., Molinari A., Bréchet Y. (1998). A dislocation-based model for all hardening stages in large strain deformation. Acta Mater..

[22] Song E., Andani M.T., Misra A. (2024). Quantification of grain boundary effects on the geometrically necessary dislocation density evolution and strain hardening of polycrystalline mg 4Al using in situ tensile testing in scanning electron microscope and HR-EBSD. J. Magnes. Alloys.

[23] Gao S., Li Z., Van Petegem S., Ge J., Goel S., Vas J.V., Luzin V., Hu Z., Seet H.L., Sanchez D.F. (2023). Additive manufacturing of alloys with programmable microstructure and properties. Nat. Commun..

[24] Fleck N.A., Hutchinson J.W. (2001). A reformulation of strain gradient plasticity. J. Mech. Phys. Solids.

[25] Gudmundson P. (2004). A unified treatment of strain gradient plasticity. J. Mech. Phys. Solids.

[26] Mirzaei D. (2015). Analysis of moving least squares approximation revisited. J. Comput. Appl. Math..

[27] Noh W., Chew H.B. (2024). Dislocation descriptors of low and high angle grain boundaries with convolutional neural networks. Extreme Mech. Lett..

[28] Ban H., Peng Z., Fang D., Yao Y., Chen S. (2020). A modified conventional theory of mechanism-based strain gradient plasticity considering both size and damage effects. Int. J. Solids Struct..

[29] Sun Z.C., Wu H.L., Cao J., Yin Z.K. (2018). Modeling of continuous dynamic recrystallization of al-zn-cu-mg alloy during hot deformation based on the internal-state-variable (ISV) method. Int. J. Plast..

[30] Chen F., Tian X., Wu G., Zhu H., Ou H., Cui Z. (2022). Coupled quantitative modeling of microstructural evolution and plastic flow during continuous dynamic recrystallization. Int. J. Plast..

[31] Edalati K., Wang Q., Enikeev N.A., Peters L.-J., Zehetbauer M.J., Schafler E. (2022). Significance of strain rate in severe plastic deformation on steady-state microstructure and strength. Mater. Sci. Eng. A.

[32] Kim H., Chang K. (2020). Triple-junction morphology classification and dihedral angle distribution during 2D grain growth. Results Phys..

[33] Tóth L.S., Estrin Y., Lapovok R., Gu C. (2010). A model of grain fragmentation based on lattice curvature. Acta Mater..

[34] Allain-Bonasso N., Wagner F., Berbenni S., Field D.P. (2012). A study of the heterogeneity of plastic deformation in IF steel by EBSD. Mater. Sci. Eng. A.

[35] Renk O., Hohenwarter A., Edalati K., Kapp M.W. (2024). Saturation of grain fragmentation upon severe plastic deformation: Fact or fiction?. Adv. Eng. Mater..

[36] Tian X., Chen F., Jiang J., Wu G., Cui Z., Qian D., Han X., Wang B., Wang H., Wang H. (2022). Experimental analyses and numerical modeling of the microstructure evolution of aluminum alloy using an internal state variable plasticity-based approach coupled with the effects of second phase. Int. J. Plast..

[37] Huang J., Xu J., Guan B., Fu R., Hu Q., Liu W., Hu Z. (2025). Effect of al addition on mechanical and corrosion behavior of B10 alloy. J. Mater. Res. Technol..

[38] Liang C., Wang N., Chen Y., Jiang C., Wu G., Zhao Q., Zhu L., Luo J. (2023). Transition of low and high-angle grain boundaries during strain rate-induced dislocation storage and annihilation. Mater. Charact..

[39] Abaray L., Flipon B., Durand M., Bayona Carrillo N., Bernacki M. (2026). Characterization and modeling of continuous dynamic recrystallization (CDRX): Application to 2139 aluminum alloy. Acta Mater..

[40] Atefi S., Parsa M.H., Ahmadkhaniha D., Zanella C., Jafarian H.R. (2022). A study on microstructure development and mechanical properties of pure copper subjected to severe plastic deformation by the ECAP-conform process. J. Mater. Res. Technol..

